# Ultra-thin strut cobalt chromium bare metal stent usage in a complex real-world setting. (SOLSTICE registry)

**DOI:** 10.1007/s12471-014-0629-6

**Published:** 2015-01-07

**Authors:** M. J. Suttorp, P. R. Stella, J. Dens, J. M. McKenzie, K. S. Park, P. Frambach, on behalf of the SOLSTICE investigators

**Affiliations:** 1 Department of Interventional Cardiology, St. Antonius Hospital, Nieuwegein, the Netherlands; 2 Department of Cardiology, University Medical Center Utrecht – Utrecht University, Utrecht, the Netherlands; 3 Department of Interventional Cardiology, Hospital Oost-Limburg, Genk, Belgium; 4 DISA Vascular, Cape Town, South Africa; 5 Strait Access Technologies, Cape Town, South Africa; 6 Institut National de Chirurgie Cardiaque et de Cardiologie Interventionnelle, Luxembourg, Luxembourg

**Keywords:** Bare metal stent, Cobalt chromium stent, Stent design

## Abstract

**Aim:**

To report clinical follow-up at 6 months after implantation of the ultra-thin strut cobalt chromium SolarFlex stent in a real-world setting.

**Methods and results:**

Patients (*n* = 240) with single or multiple vessel coronary artery disease undergoing percutaneous coronary intervention (PCI) at four sites in Europe were enrolled in the SOLSTICE (SolarFlex Stent in Routine Clinical Practice) registry. Follow-up at 6 months was 100 %. Diabetes was present in 29 % of the patients, 30 % presented with acute myocardial infarction and 17 % had unstable angina. Of the patients, 27 % had previously undergone PCI or coronary artery bypass surgery. Lesion complexity was high (50 % B2 + C type lesions). Device success was achieved in 99.7 % of cases and the major adverse cardiac event (MACE) rate was 5.8 % at 6 months of follow-up. Target lesion revascularisation (TLR) was 5.0 % at 6 months.

**Conclusions:**

The SOLSTICE registry showed that in a complex real-world setting the SolarFlex bare metal stent, with ultra-thin struts and customised scaffolding, provided low clinical MACE and TLR rates. These results provide support for the use of the latest generation bare metal stent in contemporary European practice.

## Introduction

Current clinical trials have shown that bare metal stents (BMS) are generally associated with restenosis rates of between 10 and 15 %. [1–3]. Drug-eluting stents (DES) have been widely adopted as the device of choice for the treatment of coronary artery disease (CAD) and have proved to be more efficacious than BMS, with clinical restenosis rates dropping to below 10 % [4]. However, many DES systems are limited in terms of flexibility and deliverability, increased costs relative to BMS, and the continued risk of late and very late stent thrombosis [5–7].

While DES are inherently less prone to restenosis than BMS, ongoing developments in understanding the interplay between stent design and clinical outcomes provide scope for narrowing the efficacy gap between BMS and DES, especially in larger vessels where the restenosis rates of DES and BMS already begin to converge [8]. Clinical studies such as the ISAR STEREO and ISAR STEREO II trials have demonstrated the significant advantage of thinner stent struts in reducing restenosis rates [1, 2], and first-generation cobalt chromium (Co-Cr) stents allowed stent designs with much thinner struts compared with the older stainless steel stents. Early generation Co-Cr stents had a strut thicknesses of 81–91 μm. While these dimensions likely conferred some efficacy benefits, a number of studies [1, 2, 6, 9, 10] have shown that optimal strut dimensions should remain below 75 μm in order to ensure rapid endothelialisation of the implant, which in turn is believed to decrease neointimal proliferation.

The primary aim of this post-market, non-randomised, multi-centre, prospective SOLSTICE (SolarFlex Stent in Routine Clinical Practice) registry was thus to assess the safety and efficacy of an ultra-thin strut L605 Co-Cr bare metal stent, the SolarFlex, in a real-world setting.

## Methods

### Data collection and monitoring

The study protocol was approved by the Ethics Committee at each site and each patient provided written informed consent. Patient demographics, presenting conditions, risk factors and procedural outcomes were recorded on Case Report Forms (CRF) at baseline and all major adverse cardiac events (MACE), were recorded in-hospital and at follow-up. CRFs were monitored and reviewed by an independent Clinical Research Associate (CRA) and all reported MACE were reviewed by an independent arbitrator.

### Patient population

Patients older than 18 years of age, with lesion lengths of 25 mm or less, where a maximum of three de novo or (non-stented) restenotic lesions could be planned to each be fully covered by a single stent, were included in the registry. The following exclusion conditions applied: refractory lesions that could not be pre-dilated at 20 atm; cardiogenic shock; known contraindications or allergy to aspirin, antiplatelet treatment or the stent material; did not qualify for treatment with the necessary concomitant medication; pregnancy; left main disease; history of previous in-stent restenosis; implantation of a drug-eluting stent in the study artery during the same procedure.

Initially 243 patients provided consent but according to the completed CRFs three patients did not meet all the inclusion and exclusion criteria and thus data were collected for the 240 patients who did fully met the criteria.

Balloon angioplasty and stent implantation were performed according to standard clinical practice in the participating centres and the recommendations of the SolarFlex Instructions for Use. Criteria for whether to implant a BMS or a DES were based on the current practices of each particular site. It was recommended that an appropriate dual antiplatelet regimen should be strictly adhered to for at least 30 days after the intervention.

### The SolarFlex stent system

**Fig. 1:**
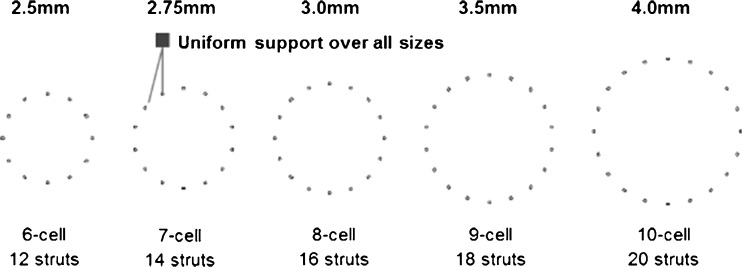
Schematic illustration showing the uniform distance between stent struts over all five stent sizes

The SolarFlex stent system consists of a cylindrical, electropolished, Co-Cr corrugated ring balloon-expandable stent mounted on a rapid-exchange coronary stent delivery catheter. The stent has an ultra-thin strut thickness down to 65 μm. Five different stent cellular configurations are used to span the five diameters of the full range, as illustrated in Fig. 1 (stents range from 12 struts around the circumference for 2.5 mm stents to 20 struts around the circumference for 4 mm stents). The stent system was available in 2.5, 2.75, 3.0, 3.5 and 4.0 mm diameters and 11, 14, 17, 20, 24 and 28 mm lengths. The stent has a low crimped profile down to 0.84 for 2.5 mm stents.

### Definitions

Clinically driven target lesion revascularisation (cTLR) was defined as any repeat percutaneous intervention of the study stent or bypass surgery of the target vessel due to symptoms or functional evidence of ischaemia in the presence of a percent diameter stenosis ≥50 %, or percent diameter stenosis of ≥70 % even in the absence of ischaemic signs or symptoms.

MACE was defined as the composite of cardiac death, myocardial infarction (MI) attributable to the target vessel, and cTLR. Any deaths due to proximate cardiac cause, unwitnessed death and death of unknown cause, and all procedure-related deaths, including those related to concomitant treatment, were classified as cardiac death. MI was defined as the presence of elevated troponin T or troponin I greater than three times the upper limit of normal. Stent thrombosis (definite or probable) was defined using the Academic Research Consortium definition [11].

Device success was defined as the ability to reach and cross the target lesion, deploy the stent and withdraw the stent delivery catheter, with the attainment of a post-stent diameter residual stenosis ≤30 % with ≥ TIMI 2 flow with the treatment device alone; standard pre-dilation and post-dilation catheters could be used. Procedural success was defined as device success with freedom from in-hospital MACE.

### Follow-up

All patients were followed up for 6 months after treatment either by clinical examination by the investigator or a telephone interview. Interviews were used to evaluate the occurrence of cTLR and MACE within the follow-up period.

### Statistical analysis

Continuous variables were presented as mean ± standard deviation and categorical variables were presented as counts and percentages.

## Results

### Patient demographics

Four European centres enrolled 240 patients who met all the inclusion and exclusion criteria in the period from April 2011 to January 2012. The average age of the patients was 66 years, with the majority being male (74 %). Over one-quarter of patients (29 %) were diabetic. A notably large number (30 %) of patients presented with acute MI (AMI). The full patient demographics and baseline clinical characteristics are given in Table 1.

**Table 1:** Baseline clinical and angiographic characteristics

Number of patients	240
Age (years)	66 ± 12.6
Male (%)	178 (74 %)
Baseline angina status	
Asymptomatic	7 (2.9 %)
Silent ischaemia	12 (5.0 %)
Stable	108 (45.0 %)
Unstable	40 (16.7 %)
AMI	73 (30.4 %)
History of	
MI	67 (27.9 %)
CABG	16 (6.7 %)
PCI	71 (29.6 %)
Risk factors	
Diabetes mellitus type 1	38 (16 %)
Diabetes mellitus type 2	31 (13 %)
Hypertension	138 (58 %)
Cholesterol	142 (59 %) [*n* = 239]^a^
Family history	114 (48 %) [*n* = 237]^a^
Smoking	186 (22 %) [*n* = 238]^a^
Number of lesions	292
Target lesion classification	
A	50 (17.1 %)
B1	92 (31.5 %)
B2	110 (37.7 %)
C	37 (12.7 %)
Unknown	3 (1.0 %)
Vessels treated	
Heft anterior descending	106 (36.3 %)
Right coronary artery	108 (37.0 %)
Circumflex	74 (25.3 %)
Saphenous valve graft	3 (1.0 %)
Ramus	1 (0.3 %)
Angulation	
< 45°	236 (80.8 %)
≥ 45°	47 (16.1 %)
≥ 90°	7 (2.4 %)
Unknown	2 (0.7 %)
Tortuosity	
Low	186 (63.7 %)
Moderate	86 (29.5 %)
High	18 (6.2 %
Unknown	2 (0.7 %)
Complexities	
Bifurcation	35 (12.0 %)
Ostial	15 (5.1 %)
Thrombus	32 (11.0 %)
Calcification	77 (26.4 %)
Diffuse disease	32 (11.0 %)
Involves side branch	12 (4.1 %)

^a^Where data were not available for all patients the number of patients with data is indicated (n)

### Angiographic characteristics

The baseline angiographic characteristics are shown in Table 1. The vessels treated were primarily the left anterior descending artery (36 %) and right coronary artery (37 %). Significantly, over 50 % of lesions were categorised as complex (B2 + C type) according to the modified American College of Cardiology (ACC) and the American Heart Association (AHA) classification [12].

### Procedural characteristics

In total 292 lesions were treated with 293 stents, representing 1.2 stents per patient. Pre-dilatation was performed in 60 % of the lesions while post-dilatation was carried out in 21 % of the lesions. The reason for stenting was primarily elective (90 %). The average stent diameter used was 3.15 mm with an average length of 17.47 mm. All devices reached the target lesion and a 99.7 % device success rate was achieved. The procedural success rate was 97.5 %. Procedural data are recorded in Table 2.

**Table 2:** Clinical outcomes

Number of stents	293
Average stent dimensions	
Diameter (mm)	3.15 ± 0.38
Length (mm)	17.47 ± 4.82
Predilation	176 (60.1 %)
Direct stenting	117 (39.9 %)
Post-dilation	60 (20.5 %)
Reason for stenting	
Elective	265 (90.4 %)
Dissection	23 (7.8 %)
Other	5 (1.7 %)
Delivery success (stents reaching lesion)	293 (100 %)
Device success (per stent)	292 (99.7 %)
Procedural success (per patient)	234 (97.5 %)
Follow-up	240 (100 %)
In-hospital events	
Total MACE	5 (2.1 %)
Cardiac death	0
Myocardial infarction	1 (0.4 %)
Target lesion revascularisation	4 (1.7 %)
Stent thrombosis (definite)	3 (1.3 %)
6-month outcome (cumulative)	
Total MACE	14 (5.8 %)
Cardiac death	1 (0.4 %)
Myocardial infarction	3 (1.2 %)
Target lesion revascularisation	12 (5.0 %)
Stent thrombosis (probable)	1 (0.4 %)
Stent thrombosis (definite)	4 (1.7 %)

### Clinical follow-up

Follow-up at 6 months was 100 % The cumulative rate of cTLR at 6 months was 5.0 %. A single case of cardiac death occurred before 30 days which, by definition, was attributed to a probable stent thrombosis. This patient was a 77-year-old male smoker with a previous history of MI who presented with unstable angina and who died of unknown causes 1 week after PCI. A second patient died of non-cardiac related causes (thus not considered a MACE). Total MACE at 6 months was 5.8 %.

## Discussion

While DES continue to be more efficacious than BMS, patients treated with BMS for such reasons as limited financial resources or contraindication to dual antiplatelet therapy still have excellent outcomes in modern clinical practice. This is demonstrated through ongoing BMS registries and meta-analyses and thus continues to warrant further study of BMS [13].

A review of all multi-centre registries or clinical trials of single Co-Cr stent types with more than 100 patients and 6–9 month follow-up was carried out to analyse the results from the SOLSTICE study in the context of other Co-Cr bare metal stents. (Note that studies restricted to patients presenting with STEMI were also excluded from this review). A comparison of the key results of these studies is shown in Table 3.

**Table 3:** Data from a review of all six- to nine-month multi-centre registries or clinical trials of single Co-Cr stent types with more 100 patients (MEDLINE search results extend to 31 May 2013; table excludes studies restricted to patients presenting only with STEMI)

Registry	Stent name	Material	Strut thickness (μm)	Number of patients	Average stent length (mm)	Average stent diameter (mm)	Presenting conditions	Follow-up (months)	Delivery success	MACE	TLR/TVR^a^
MULTIBENE [22]	PRO-Kinetik	SiC-coated L605	60–120	198	15.7 ± 3 .5	3.21 ± 0.45	No AMI	6	–	11.4 %	7.6 %
Arthos Pico Austrian Multicentre [23]	Arthos Pico	Phynox Co-Cr	65	203	16.2 ± 6.5	2.58 ± 0.22	Real-world	6	98.0 %	13.0 %	6.0 %
SOLSTICE	SolarFlex	L605 Co-Cr	65	240	17.5 ± 4.82	3.15 ± 0.38	Real-world	6	100 %	5.8 %	5.0 %
CoroFlex Blue registry [24]	CoroFlex Blue	L605 Co-Cr	65	2315	15.6 ± 4.4	3.01 ± 0.43	Real-world	6	98.5 %	9.2 %	5.5 %
MILES [25]	Skylor	L605 Co-Cr	70–95	1020	19.5 ± 10.3	3.03 ± 0.43	Real-world	6+	98 %	9.9 %	5.0 %
VISION registry [17]	Vision	L605 Co-Cr	81	267	17.2 ± 6.3	3.34 ± 0.37	No AMI	6	100 %	6.2 %	4.3 %
VIVE [16]	Vision	L605 Co-Cr	81	429	–	–	Real-world	6	99.3 %	6.8 %	1.4 %
RISICO [18]	Vision	L605 Co-Cr	81	143	17.0 ± 3.9	2.41 ± 0.14	No AMI	6	96.7 %	11.6 %	5.8 %
Driver registry [26]	Driver	MP35N Co-Cr	91	298	–	–	Real-world	9	100 %	8.4 %	7.0 %
CLASS [27]	Driver	MP35N Co-Cr	91	202	–	–	No AMI	6	100 %	12.4 %	9.4 %

^a^Where available as an endpoint, cTLR data were been used preferentially for comparison purposes

Randomised trials have demonstrated that BMS with thin struts result in lower restenosis rates than thick-strut stents [1, 2]. The current understanding of stent design has also demonstrated the advantages of greater scaffolding support [14]. This is supported by computational fluid dynamics which has shown that increasing the number of struts around the circumference results in a smaller intrastrut area exposed to low wall shear stress [15]. Increased scaffolding comes at the expense of increased metal-to-artery contact, which is thought to exacerbate any foreign body effects of the implant, and a denser crimped configuration. Applying these principles to the SolarFlex stent, a customised scaffold structure was created where each stent diameter has its own cell design to provide uniform support over all artery sizes. The MACE data, while often difficult to compare between studies because of varying definitions, reveal the SolarFlex stent to have a very low MACE rate (5.8 %) compared with the other studies detailed in Table 3, a fact which may be attributable to the application of these stent design principles. In addition, the cumulative TLR results for the study (5.0 %) compare favourably with other studies in the selection (bar that of the study of the Vision stent carried out by Xu et al. [16] which appears to have an atypical cTLR rate of 1.4 % when compared with the other studies of this same stent carried out by Kereiakes et al. [17] and Brambilla et al. [18] which have TLR rates of 4.3 and 5.8 % respectively. It should be noted that the mean stent diameter in our study was above 3.0 mm and the mean stent length was below 20 mm, which predict relatively low rates of restenosis. The sizes of the SolarFlex in the study do, however, fall within the same range as the other studies.

While substantially more data have been collected on L605 Co-Cr stents that other Co-Cr alloys, choosing L605 as BMS material may provide clinical advantages. The average TLR rate of the L605 alloy stents is 4.5 % compared with that of 8.5 % for the MP35N alloy, with average MACE rates being 8.3 and 10.4 % respectively. The single study using the Phynox alloy demonstrated a TLR rate of 6 % and a MACE rate of 13 %.

While DES tend to suffer from limited deliverability and flexibility (due to the presence of coatings, less firm crimping procedures, and the use of earlier generation BMS platforms) Co-Cr BMS have a high delivery success rate as shown for all the studies listed in Table 3. Data relating to the Abbott Vascular (formerly Guidant) Multilink family of stents provides an interesting case study to demonstrate the evolution of deliverability for Co-Cr stents to parallel improved clinical data for its Co-Cr Multilink Vision stent. A decade ago, Kereiakes et al. [17] showed, for this family of stents, that late lumen loss is related to strut thickness, and that the relative biocompatibility of Co-Cr is also supported by this data. Interestingly, as Ormiston et al. show [19], a steady reduction in crossing profile has been linked to improved deliverability, with the early generation Multilink stent having a crossing profile of 1.53 mm showing worse trackability than the Multilink Duet, which has a crossing profile in the order of 1.17 mm, despite both systems having similar flexibility. The continued pursuit of improved deliverability has thus driven profiles for commercial stents down even further to the order of 1 mm [20], with the SolarFlex 3.5 mm delivery system having a crimped profile of 0.98 mm. A relatively high direct stenting rate in the complex real-world setting of this SOLSTICE study of 39.9 %, together with a delivery success rate of 100 %, supports this evidence for efficient deliverability of a BMS ultra-thin strut Co-Cr system. The technological improvements related to BMS deliverability have kept pace with improvements in clinical outcome.

Four cases of definite stent thrombosis were recorded during the SOLSTICE study and an interrogation of these cases reveals that all four of the patients presented with AMI. While the mechanism of stent thrombosis is not limited to a single factor (various factors related to the procedure, lesion and patient are relevant), it is known that patients presenting with AMI have an increased risk of stent thrombosis [21].

## Study limitations

A lack of angiographic follow-up can represent a study limitation; however, clinical endpoints, as used in the SOLSTICE study, are possibly of more relevance for assessment of patient outcomes. Although lack of randomisation is a limitation, the study sought to examine real-world clinical safety and effectiveness in a milieu of proven DES superiority. Approximately 2840 PCIs were performed in the four centres over the study enrolment period, while only 240 patients were enrolled in the registry; selection bias is therefore possible.

## Conclusions

Positive developments in bare metal stent material and design understanding continue to be seen through the improving clinical data being gathered in real-world registries. Judicious use of advanced bare metal stent designs can result in suitable clinical outcomes, as is suggested in this study by the low MACE and TLR rates of an ultra-thin strut L605 cobalt-chromium bare metal stent with a customised cell design providing uniform support over all artery sizes in the stent range.
